# Artemisinin Targets Transcription Factor PDR1 and Impairs *Candida glabrata* Mitochondrial Function

**DOI:** 10.3390/antiox11101855

**Published:** 2022-09-20

**Authors:** Pan Zhu, Chaoping Yue, Xin Zeng, Xiulai Chen

**Affiliations:** 1 State Key Laboratory of Food Science and Technology, Jiangnan University, Wuxi 214122, China; 2 State Key Laboratory of Membrane Biology, School of Life Sciences, Tsinghua University, Beijing 100084, China; 3 Anhui Province Key Laboratory of Pollutant Sensitive Materials and Environmental Remediation, Huaibei Normal University, Huaibei 235000, China

**Keywords:** *Candida glabrata*, artemisinin, PDR1, mitochondria

## Abstract

A limited number of antifungal drugs, the side-effect of clinical drugs and the emergence of resistance create an urgent need for new antifungal treatment agents. High-throughput drug screening and in-depth drug action mechanism analyzation are needed to address this problem. In this study, we identified that artemisinin and its derivatives possessed antifungal activity through a high-throughput screening of the FDA-approved drug library. Subsequently, drug-resistant strains construction, a molecular dynamics simulation and a transcription level analysis were used to investigate artemisinin’s action mechanism in *Candida glabrata*. Transcription factor pleiotropic drug resistance 1 (PDR1) was an important determinant of artemisinin’s sensitivity by regulating the drug efflux pump and ergosterol biosynthesis pathway, leading to mitochondrial dysfunction. This dysfunction was shown by a depolarization of the mitochondrial membrane potential, an enhancement of the mitochondrial membrane viscosity and an upregulation of the intracellular ROS level in fungi. The discovery shed new light on the development of antifungal agents and understanding artemisinin’s action mechanism.

## 1. Introduction

Fungal pathogens causing life-threatening systemic infections remain an underestimated threat to global public health, which cause 1.5 million people being infected every year [1,2]. Candidiasis is one of the major fungal diseases, which is mainly caused by *Candida albicans* and *Candida glabrata* [3,4,5]. The candida infections occur in the oral cavity, upper and lower airways, gastrointestinal and urinary tracts on wounds [6]. Psoriatic patients, cancer patients and immunocompromised persons such as human immunodeficiency virus (HIV) patients, are susceptible to fungal infections [7,8].

At present, three major classes of antifungal drugs have been clinically used for the treatment of fungal infections: azoles, polyenes and echinocandins [9]. In addition, other antifungal drugs such as pyrimidines are also used in combination therapy [10]. Azole drugs mainly include fluconazole, ketoconazole and miconazole, among which fluconazole is widely used for treating candidiasis because of its low price and few side-effects [5]. Amphotericin B is a polyene antifungal drug, which binds to the ergosterol and damages fungal cell membrane leading to cell death [11]. Echinocandins such as caspofungin can target the fungal β-1,3-d-glucan synthase of cell wall [12]. However, the side-effect and toxicity of polyene and echinocandins limit their wide clinical application, and further drug resistance to azoles are constantly emerging [13]. Therefore, there is an urgent need for developing new treatment strategies.

The development of an entirely new antifungal drug is costly and hard. Thus, repurposing known drugs in the Food and Drug Administration (FDA)-approved medications library holds great potential for finding new antifungal drugs [14,15,16]. FDA-approved drugs have been used to treat various diseases such as cancer, neurodegenerative, cardiovascular disease and infections, and further, these compounds have a safe toxicity profile, well-known chemical properties, pharmacokinetic properties and reported biological functions [9]. High-throughput screening of these known pharmacologically active antifungal drugs has recently gained a lot of attention [9,17,18]. These studies have mainly focused on *C. albicans*, but *C. glabrata* as the second most common species associated with candidiasis has not been well studied. 

The goal of this study was to find new anti-*C. glabrata* drugs and get insight into the mechanism of drug action. Here, we report a new antifungal drug—artemisinin—from the FDA-approved drug library by high-throughput screening, and further investigate artemisinin’s action mechanism in *C. glabrata*. Artemisinin can impair *C. glabrata*’s mitochondrial function via transcription factor pleiotropic drug resistance 1 (PDR1) to regulate the drug efflux pump and ergosterol biosynthesis pathway.

## 2. Materials and Methods 

### 2.1. Strains, Media and Chemicals

All *C. glabrata* strains used in this study are listed in Table S1. The mutant strains were constructed by standard genetic techniques under a *C. glabrata* ATCC 55 background, and knockout strains were constructed by homologous recombination following a previously described protocol [19]. Yeast strains were cultivated in YPD medium (1% yeast extract, 1% peptone, 2% glucose) or YPG medium (1% yeast extract, 1% peptone, 2% glycerol); solid media contained 1.5% agar. FDA-approved drugs used in screening antifungal drugs were purchased from Selleck (Houston, TX, USA), this library included artemisinin and its analogues artesunate, dihydroartemisinin, artemether. 

### 2.2. Screening Antifungal Drugs 

Selleck’s FDA-approved drug library was used for new antifungal drugs; this library had 2572 compounds, most compounds were dissolved in dimethyl sulfoxide (DMSO) at 10 mM stock solution, some compounds were dissolved in deionized water. *C. glabrata* was cultivated in YPD liquid medium at 180 rpm (30 °C) overnight and secondary cultured in YPD medium until the log phase. Yeast cells were harvested and diluted to an absorbance at 600 nm (OD_600_) of 0.1 in YPG medium, this suspension was added to the 96-well plates with different compounds (10 μM final concentration) at 100 µL final volume. After 12 h or 24 h of incubation at 30 °C, the absorbance at 600 nm was examined. The percentage of drug inhibition on fungi was calculated relative to the negative control (DMSO).

### 2.3. Spot Assay and Growth Assay

To analyze the artemisinin sensitivity of *C. glabrata*, a spot dilution assay and growth kinetics assessments were employed. For the spot assay, yeast cells were cultured in YPD liquid medium at 180 rpm (30 °C) overnight, and a secondary culture was grown until the log phase. Then, yeast cells were harvested and washed by sterile phosphate-buffered saline (PBS) buffer twice and diluted to an absorbance at 600 nm (OD_600_) of 0.5 by a sterile PBS buffer. After that, 10-fold serial dilutions of yeast cells were spotted on YPG medium plates with or without artemisinin. Agar YPG plates were incubated at 30 °C for 72 h, and photos were taken for inhibition analysis. For the growth assay, yeast cells were cultivated in YPD liquid medium at 30 °C until the log phase. Cells were washed twice by sterile PBS buffer and resuspended in YPG medium to an initial OD_600_ of 0.1. Yeast cells were then cultured with or without artemisinin at 180 rpm (30 °C), and OD_600_ was measured at different incubation times such as 10 h and 20 h to analyze artemisinin inhibition.

### 2.4. Colony-Forming Units Counting Assay

*C. glabrata* strains were first cultured in liquid YPD medium at 30 °C overnight and we harvested yeast cells. Cells were resuspended in YPG medium at 0.1 initial OD_600_ in the presence of artemisinin or DMSO (negative control) and incubated at 180 rpm (30 °C) for 10 h. Yeast cells were collected by centrifugation at 3500 rpm for 5 min and pellets were diluted with YPD medium to obtain serial gradient dilutions. A quantity of 100 µL of yeast suspension was inoculated on solid YPD medium containing 1.5% agar, and the plates were cultured at 30 °C. After a 48 h incubation, photos of the plates were taken by a camera to count the colony number and measure the colony size.

### 2.5. Fungal RNA Isolation

Yeast cells were grown in YPD liquid medium at 30 °C overnight, and cells were harvested and washed by sterile PBS buffer. Then, yeast cells were resuspended in YPG liquid medium and cultured at 30 °C until the log phase. Yeast cells were incubated with or without artemisinin at 30 °C for 20 min or 2 h. After that, cells were collected by centrifugation at 3500 rpm at 4 °C for 5 min, washed three times by ice-cold water and resuspended in Trizol (Invitrogen, Grand Island, NY, USA). The total RNA was extracted by a MiniBest universal RNA extraction kit (TaKaRa Bio, Shiga, Japan). The RNA quantity was determined by the NanoDrop 2000 spectrophotometer.

### 2.6. Expression Analysis by Quantitative Real-Time PCR

A quantity of 1 μg of total RNA was used for the cDNA synthesis using a PrimeScript II first-strand cDNA synthesis kit (TaKaRa Bio, Shiga, Japan). The quantification of the gene expression profile was evaluated by SYBR Premix *Ex Taq* (TaKaRa Bio, Shiga, Japan), and the housekeeping gene cgACT1 was used to normalize the gene expression as a standard control. Primers used in this study are listed in Table S2. All experiments were done in independent biological triplicate.

### 2.7. Measurement of ROS Levels

ROS production was measured by the oxidant-sensitive probe dichlorofluorescin diacetate (DCFH-DA). Yeast cells were cultured in liquid YPD medium overnight and harvested by centrifugation. Then, cells were washed by sterile PBS buffer for twice, cells were resuspended in YPG medium and grown at 30 °C until the log phase. After that, yeast cells were cultured with or without artemisinin for 2 h, cells were harvested and resuspended in PBS with DCFH-DA at 2 μM final concentration. Cells were incubated with DCFH-DA for 60 min and measured by a FACSCalibur flow cytometer at 495 nm excitation and 529 nm emission. Finally, FlowJo 10.6.1 software (Becton, Dickenson and Company, Ashland, OR, USA) was used for analyzing ROS level. 

### 2.8. Determination of Mitochondrial Membrane Potential

The mitochondrial membrane potential in *C. glabrata* was measured by examining the fluorescence intensity of rhodamine 123 (Rh123). Yeast cells were cultivated in YPD liquid medium overnight and a secondary culture was grown in YPG liquid medium until the log phase after being washed by a sterile PBS buffer. Then, cells were harvested by centrifugation and resuspended in YPG medium with or without artemisinin for 2 h. After that, yeast cells were incubated with 2 μM final concentration Rh123 for 30 min at 30 °C, then washed and resuspended in PBS buffer. The mitochondrial membrane potential was measured by a Nikon Ti-E confocal microscope with the excitation wavelength at 488 nm and the emission spectrum at 500–550 nm. 

### 2.9. Mitochondrial Isolation and Membrane Viscosity Assessment

Mitochondrial isolation was performed as previously described [20,21]. Yeast cells were cultured in YPD liquid medium overnight at first and then in YPG medium with or without artemisinin. Cells were harvested and washed by distilled water and DTT buffer (0.1 M Tris, 10 mM DTT). Mitochondria were isolated from *C. glabrata* by Zymolyase digestion, and harvested cells were resuspended in Zymolyase buffer (20 mM KH_2_PO_4_, 1.2 M sorbitol) containing 7 mg Zymolyase per gram wet weight. Cells were homogenized in a mitochondrial isolation buffer (5 mM HEPES, 70 mM sucrose, 0.22 M mannitol) containing 0.2% (wt/vol) BSA on ice. The homogenate was centrifuged at 800× *g*, 4 °C for 10 min to remove cell debris and nuclei. The pellet was discarded, and the supernatant was centrifuged at 10,000× *g*, 4 °C for 10 min to obtain the mitochondrial fraction. The final pellets were resuspended in a mitochondrial isolation buffer (no BSA), and the BCA Protein assay kit was used for the protein quantification. The mitochondrial membrane viscosity was assessed as previously described [22]. In brief, 200 mg of fresh isolated mitochondria from yeast cells treated with artemisinin or DMSO (negative control) was equilibrated with 100 nM final concentration MC540 in an isolation buffer at 30 °C for 5 min. The fluorescence intensity of MC540 was examined by a microplate reader (ThermoFisher, Waltham, MA, USA) with the excitation at 485 nm and the emission at 590 nm.

### 2.10. Molecular Dynamics Simulation

To investigate the mechanism of the decreased artemisinin sensitivity in PDR1 mutants, molecular dynamics simulations were performed for both the wild type PDR1 and mutant PDR1 with artemisinin following a previously described method [23]. Tleap module in Amber14 was used for building the force field parameter for each system, and the force field parameters were generated through ff14SB and GAFF2 force fields. Every simulation system was solvated in a TIP3P water box, for the solvent environment, and a buffer distance of 10 Å was applied. To neutralize each system, sodium ions were added. The energy minimization was performed in two steps, using the steepest descent and the conjugate gradient methods to remove undesirable atomic contacts. The method of particle mesh Ewald was applied to compute the long-range electrostatic interactions and the van der Waals interactions were treated with a switching function for smoothly turning off. The system was minimized for 20,000 steps and heated gradually from 0 K to 300 K for 300 ps under the weak constraint of 10 kcal (mol Å2)^−1^. After that, the NPT ensemble was used for the followed equilibration and MD simulation. The molecular mechanics Poisson–Boltzmann surface area approach was applied to estimate the substrate binding affinity. VMD was used for generating the visualization and figures of protein structures.

### 2.11. Statistical Analysis

The results were analyzed by GraphPad Prism 6 and Origin 8.5 software. A comparison was performed by a one-way analysis of variance (ANOVA) or unpaired two-tailed Student *t* test, and *p*-value < 0.05 was considered to be statistically significant. Statistical details are indicated in the figure legends.

## 3. Results

### 3.1. High-Throughput Screening Identifies New Antifungal Drugs

**Figure 1:**
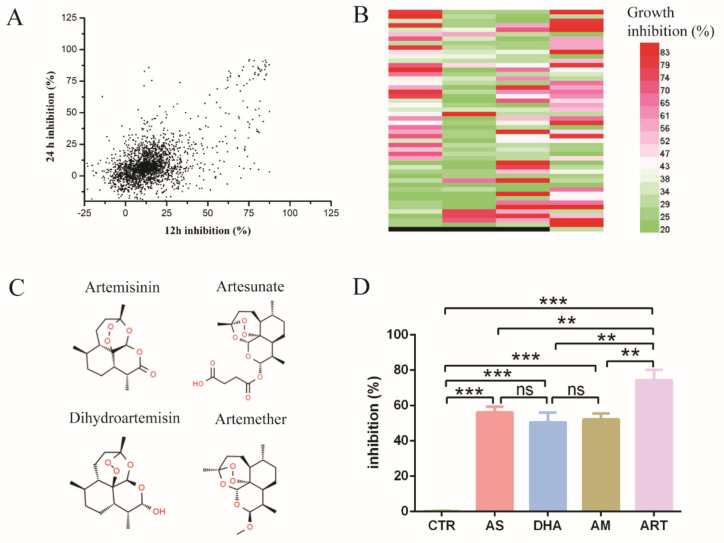
High-throughput screening for new antifungal drugs. (**A**) The inhibition rate of Selleck’s FDA-approved drugs effect on C. glabrata ATCC 2001. *X*-axis means inhibition rate measured at 12 h, *Y*-axis means inhibition rate measured at 24 h. The concentration of drugs was 10 μM. (**B**) Drugs had antifungal activity both at 12 h and 24 h, as confirmed by three independent experiments. Different colors mean different levels of inhibition rate; black means empty (no drug). Each band denotes an individual drug; total number of drugs was 197. (**C**) Chemical structure of artemisinin and derivatives, including artemisinin, dihydroartemisinin, artesunate and artemether. (**D**) The inhibition rate of artemisinin and derivatives: artemisinin had the most obvious effect on the growth of C. glabrata. CTR: control, AS: artesunate, DHA: dihydroartemisinin, AM: artemether, ART: artemisinin. Data are mean ± SD of three independent experiments. The data were analyzed by GraphPad Prism. ** *p*-value < 0.005, *** *p*-value < 0.0005.

Fungal pathogens threaten human health, in which the genus *Candida* is predominant. Due to the toxicity of antifungal drugs and the emergence of resistant strains, new treatment strategies are urgently needed. In this study, Selleck’s FDA-approved drug library was used to find new antifungal drugs. We identified inhibitors of *C. glabrata*’s growth from 2572 known pharmacokinetic properties compounds. First, we measured the drugs’ inhibition on *C. glabrata* by measuring the absorbance at 600 nm after 12 h and 24 h of incubation and found that many drugs had antifungal activity (Figure 1A). These selected drugs included five classes of known antifungal agents: polyenes such as amphotericin B, echinocandins such as caspofungin acetate, imidazoles such as bifonazole, triazoles such as fluconazole and antiseptics (Table 1). These results indicated that the high-throughput drug screening strategy was effective for finding new antifungal drugs from the FDA-approved drug library. However, a few drugs improved the growth of *C. glabrata*, which might be useful for industrial application of *C. glabrata* for the production of chemicals.

Then, we chose the drugs with an inhibition rate more than 20% both at 12 h and 24 h in Figure 1A and tested their antifungal activity by three independent experiments (Figure 1B). The number of drugs was up to 197 species, among which some kind of sesquiterpene drugs caught our attention. These drugs were artemisinin and its derivatives, which were widely known as antimalarial drugs (Figure 1C). Artemisinin and all its derivatives in Selleck’s FDA-approved drug library had antifungal activity, among which artemisinin (ART) had the highest inhibition rate of up to 74.33 ± 5.85%. In addition, the inhibition rate of artesunate (AS), artemether (AM) and dihydroartemisinin (DHA) was 56.20 ± 3.08%, 52.13 ± 3.42% and 50.57 ± 5.46%, respectively (Figure 1D). DMSO as a negative control had little inhibition activity on *C. glabrata*. These results indicated that artemisinin and its derivatives are potential antifungal drugs. 

### 3.2. Artemisinin Is Active against Candida glabrata

**Figure 2:**
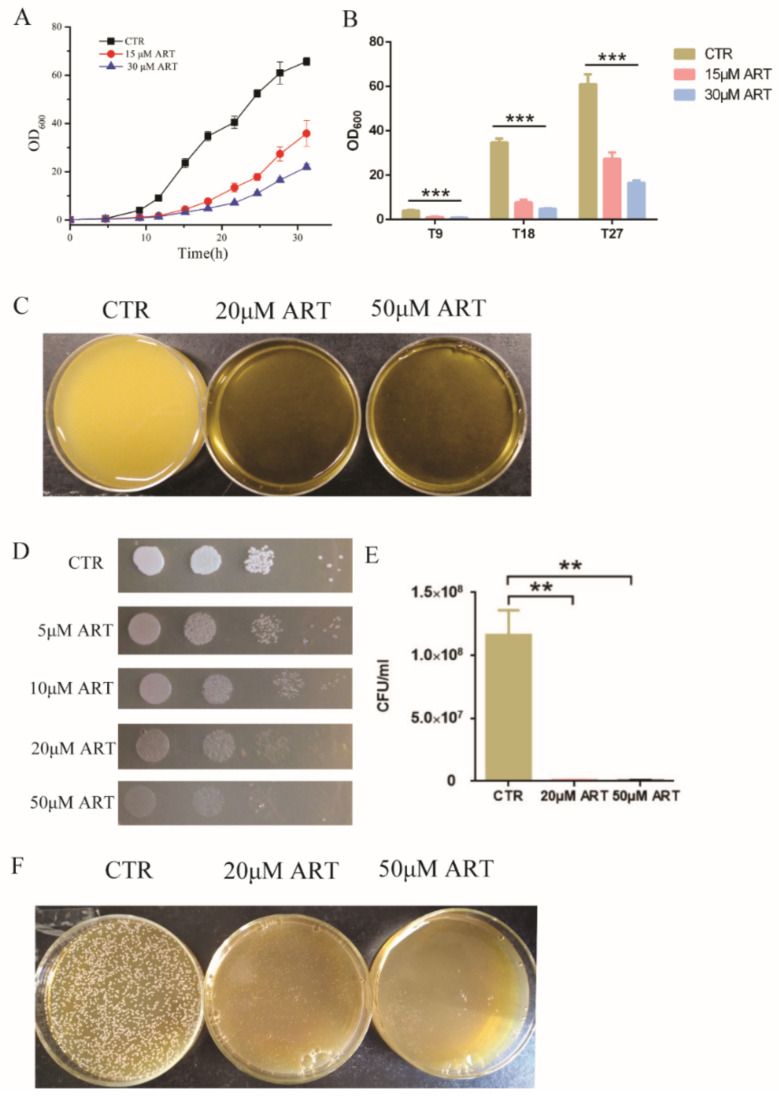
Artemisinin is active against *C. glabrata*. (**A**) The growth curve of *C. glabrata* with artemisinin. DMSO was used as a negative control (CTR). Data are mean ± SD of three independent experiments. (**B**) OD_600_ measurement after different incubation time. T9 means 9 h incubation, T18 means 18 h incubation, T27 means 27 h incubation. Data are mean ± SD of three independent experiments. Data were analyzed by GraphPad Prism, a comparison was performed by ANOVA and *p*-values were less than 0.0001. *** *p*-value < 0.0005. (**C**) Pictures taken by camera when performing artemisinin inhibition in liquid medium. (**D**) Artemisinin inhibited the growth of *C. glabrata* by spot assay. Tenfold serial dilutions of yeast cells were spotted on plates with or without artemisinin; 4 dots mean four times dilution of yeast cells. DMSO was used as a negative control (CTR); ART means artemisinin. (**E**) Artemisinin showed significant inhibition of the colony number of *C. glabrata* by counting colony forming units. Data are mean ± SD of three independent experiments. Data were analyzed by GraphPad Prism. ** *p*-value < 0.005. (**F**) Pictures taken by camera when performing artemisinin inhibition on plates.

To study the antifungal action of artemisinin and its derivatives, we chose artemisinin as a representative drug because of its highest inhibition rate and measured its effect on *C. glabrata*. First, *C. glabrata* was incubated with 15 μM and 30 μM artemisinin in liquid medium; the optical density at 600 nm (OD_600_) was measured as a readout of cell growth at different incubation times. The cell growth curves showed that artemisinin inhibited the growth of *C. glabrata* (Figure 2A). Then, we chose three incubation times (9 h, 18 h and 27 h) to conduct a statistical analysis, and the results showed that OD_600_ was decreased obviously when treated with artemisinin compared with the negative control (Figure 2B). After 9 h of incubation with 15 μM ART, OD_600_ was decreased by 72.57 ± 4.70% compared with the negative control. With the increase of artemisinin’s concentration, OD_600_ was decreased gradually. OD_600_ was decreased by 78.58 ± 1.06% in the presence of 30 μM ART compared with the negative control. In addition, when incubated with 15 μM and 30 μM artemisinin for 18 h, OD_600_ was decreased by 77.82 ± 3.38% and 86.20 ± 0.69%, respectively. After 27 h of incubation in 15 μM and 30 μM artemisinin, OD_600_ was decreased by 55.07 ± 4.76% and 72.86 ± 1.75%, respectively. Meanwhile, we cultured *C. glabrata* with 20 μM and 50 μM artemisinin in liquid medium and took photos for a visual observation (Figure 2C).

Then, we carried out a spot assay to analyze the effect of artemisinin on *C. glabrata*, and the results are shown in Figure 2D. Artemisinin inhibited the growth of *C. glabrata* on plates, and a higher concentration of artemisinin showed a more significant inhibition on *C. glabrata*’s growth. Further, fungal colony-forming units (CFU) were counted to confirm this effect. Figure 2E shows that artemisinin had a significant effect on *C. glabrata* colony forming compared with the negative control. In the presence of 20 μM and 50 μM artemisinin, the yeast colony number was decreased by 99.27 ± 0.04% and 99.51 ± 0.16%, respectively. Additionally, the yeast colony’s inhibition in the presence of artemisinin on plates was photographed by camera (Figure 2F). These results indicated that artemisinin had an effective activity on *C. glabrata*, which confirmed it was a good candidate for antifungal agents. 

### 3.3. Artemisinin Targets Transcription Factor PDR1

**Figure 3:**
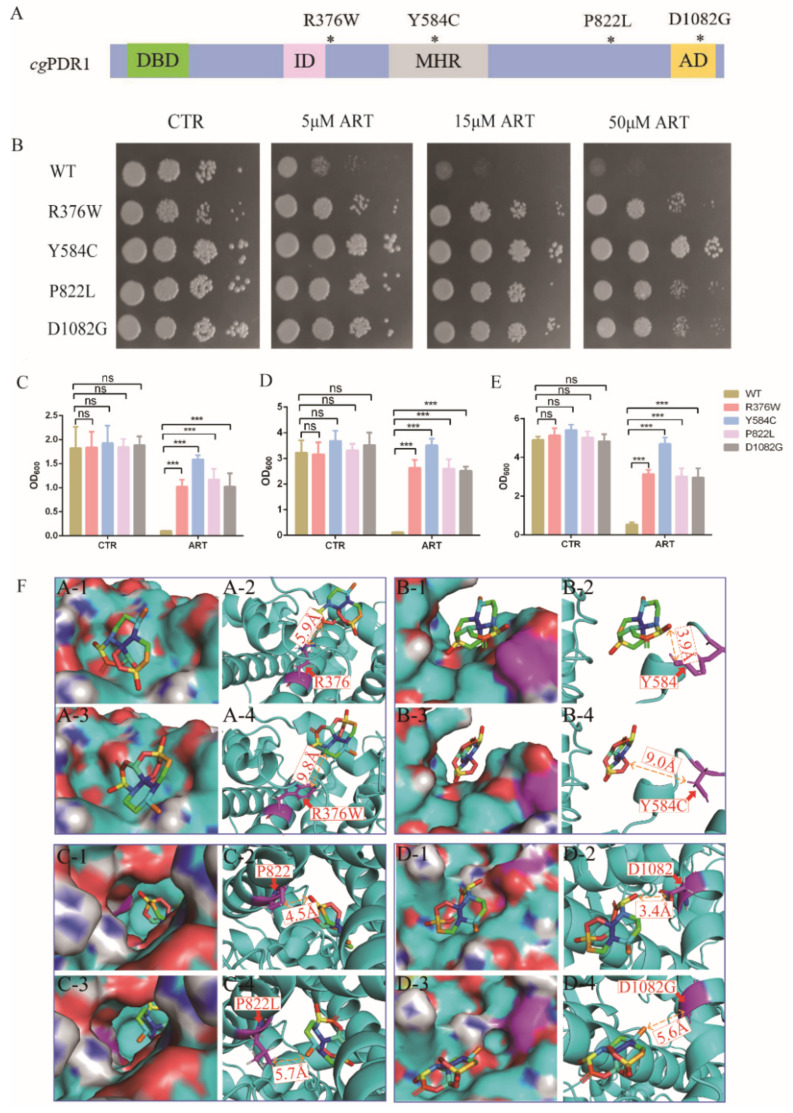
Artemisinin targets transcription factor PDR1. (**A**) Schematic showing CgPDR1 putative functional domains and mutation sites. * means the location of mutation sites; DBD, DNA-binding domain; ID, inhibitory domain; MHR, middle homology region; AD, activation domain. (**B**) PDR1 mutant strains were resistant to artemisinin by spot assay. Four dots mean 10-fold serial dilutions of yeast cells. PDR1 mutations were resistant to artemisinin in liquid medium after a 9 h incubation (**C**), 18 h incubation (**D**) and 27 h incubation (**E**). Data are mean ± SD of three independent experiments. Data were analyzed by GraphPad Prism. *** *p*-value < 0.0005; ns means no significant change. (**F**) Three-dimensional structure showing the action of artemisinin on wild-type or mutant PDR1.

To investigate the mechanism of artemisinin’s action on *C. glabrata, C. glabrata* mutants and a molecular dynamics simulation were used for searching target genes of artemisinin. Among the genes of the pleotropic drug resistance (PDR) network in *Saccharomyces cerevisiae*, transcription regulator PDR1 and its target genes were determinants of yeast resistance to artesunate, which was one of artemisinin derivatives [24]. Moreover, PDR1 mutant strains were resistant to known antifungal drugs such as fluconazole [25,26,27]. According to these previous studies, PDR1 mutant strains PDR1^R376W^, PDR1^Y584C^, PDR1^P822L^ and PDR1^D1082G^ were constructed and then used to measure artemisinin’s sensitivity (Figure 3A). We first tested the sensitivity of PDR1 mutant strains to artemisinin by a spot assay. The results showed that 5 μM artemisinin could inhibit the growth of the wild-type strain, but PDR1 mutant strains could not be inhibited even with 50 μM artemisinin (Figure 3B). Then, we analyzed the antifungal effect of artemisinin by making a comparison in growth curves between PDR1 mutant stains and the wild-type strain in the same liquid media. Wild-type and PDR1 mutant strains were incubated with 10 μM artemisinin, and then OD_600_ was measured after 9 h (Figure 3C), 18 h (Figure 3D) and 27 h (Figure 3E) of incubation time, respectively. The wild-type strain had a similar growth rate as PDR1 mutant strains with no artemisinin incubation (negative control), indicating that PDR1 mutations had no effect on the growth of *C. glabrata*. However, the wild-type strain was significantly inhibited with artemisinin incubation, and PDR1 mutant strains did not show that inhibition, indicating that PDR1 mutant strains were resistant to artemisinin. 

Further, molecular dynamics simulations were performed to investigate how these PDR1 mutations impacted artemisinin’s sensitivity in *C. glabrata*. First, a 3D model of artemisinin interacting with PDR1 was constructed by AlphaFold. Simulations of artemisinin in wild-type PDR1 or point mutant PDR1 were performed and are shown in Figure 3F. The binding free energy was calculated using the molecular mechanics Poisson–Boltzmann surface area method [28], and the results showed that the binding affinity of artemisinin with wild-type PDR1 (−21.34 kcal/mol) was stronger than that with point mutant PDR1^R376W^ (−10.04 kcal/mol) (Table 2). Similar results were also obtained in other point mutation such as Y584C, P822L and D1082G. These simulation results demonstrated that PDR1 mutations had a lower sensitivity to artemisinin than wild-type PDR1. Then, based on the catalytic mechanism, the distance between single point mutant residues (R376W, Y584C, P822L, D1082G) and artemisinin was analyzed. The distance between mutant residue R376W and artemisinin was 9.8 Å, which was longer than that of wild-type residue R376 (5.9 Å). Other mutant residues showed similar simulation results, that is, the distances between artemisinin and Y584, P822 and D1082 were 3.9 Å, 4.5 Å and 3.4 Å, respectively. They were all shorter than the distance between point mutant residues Y584C (9.0 Å), P822L (5.7 Å), D1082G (5.6 Å). From these results, we found that the distance between amino acid residues and artemisinin was partly correlated with the phenotype of the artemisinin effect. Artemisinin had no effect on the PDR1^Y584C^ mutant strain even on 50 μM artemisinin, and other mutant strains were all resistant to artemisinin compared with the wild-type strain (Figure 3B). The growth inhibition by broth assay also showed that the PDR1^Y584C^ mutant strain was more resistant to artemisinin than other mutant strains. Therefore, molecular dynamics simulations had the same trends as the phenotype experiments, suggesting that PDR1 played an important role in artemisinin’s action on *C. glabrata*.

### 3.4. Artemisinin Effects on Ergosterol Synthesis through PDR1 

**Figure 4:**
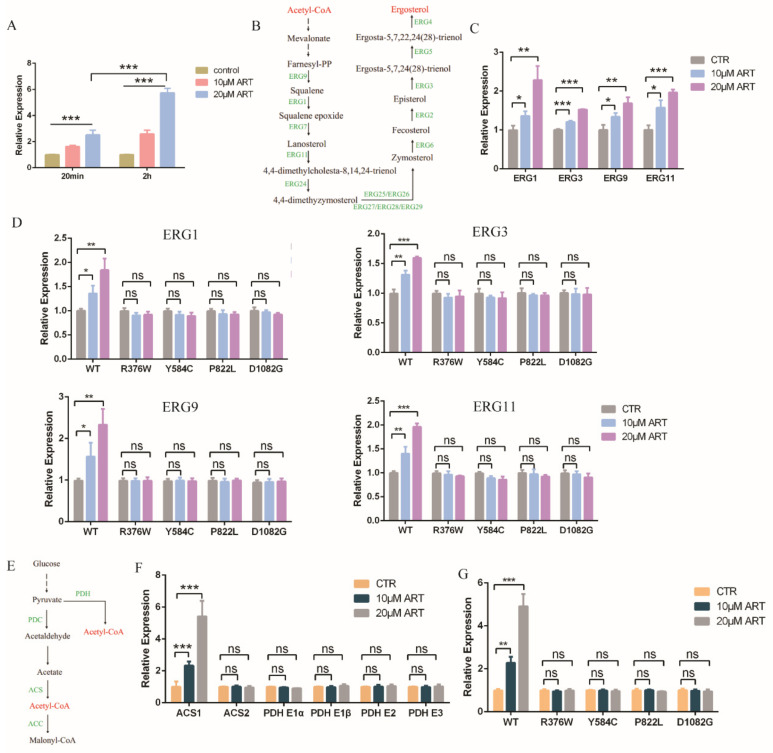
Artemisinin effects on ergosterol and acetyl-CoA synthesis through PDR1. (**A**) Transcription level of *Cdr2* with artemisinin. Incubation time were 20 min and 2 h, respectively. Data are mean ± SD of three independent experiments. Data were analyzed by GraphPad Prism. *** *p*-value < 0.0005. (**B**) Ergosterol synthesis pathway. (**C**) Genes involved in ergosterol synthesis pathway were downregulated with artemisinin. (**D**) *Erg1*, *Erg3*, *Erg9* and *Erg11* transcription levels had no significant change with artemisinin in PDR1 mutants. (**E**) Acetyl-CoA synthesis pathway. (**F**) *Acs1* but not *Acs2* or *Pdh* complex was upregulated in the presence of artemisinin. (**G**) *Acs1* transcription level had no significant change with artemisinin in PDR1 mutant strains. Data are mean ± SD of three independent experiments. Data were analyzed by GraphPad Prism. * *p*-value < 0.05, ** *p*-value < 0.005, *** *p*-value < 0.0005, and ns means no significant change (*p*-value > 0.05).

To further study how artemisinin influenced fungal cellular pathways and inhibited the growth of *C. glabrata*, we analyzed the downstream pathway of transcription factor PDR1. As one of the target genes of PDR1, the transcription level of *ScPdr5* was up 13-fold in the presence of artemisinin analogue artesunate in *S. cerevisiae* [24]. *C. glabrata* had a homologous protein named *CgCdr2*. Thus, we measured the transcription level of *Cdr2* with artemisinin incubation by a quantitative real-time PCR (qPCR) assay, and the results showed that the transcription level of *Cdr2* was upregulated (Figure 4A). *Cdr2* transcription could be further enhanced by a high drug concentration and long incubation time compared with the negative control DMSO. The homologue protein of *CgCdr2*—*CaCdr1* was related to membrane fluidity and ergosterol content in *C. albicans*, suggesting that ergosterol played an important role in membrane fluidity [29]. Hence, we wanted to know whether artemisinin had an effect on ergosterol biosynthesis pathway or not. This pathway in *C. glabrata* could convert acetyl-CoA to ergosterol (Figure 4B), similar to the cholesterol biosynthesis pathway in the mammalian cell [30]. First, we measured the gene transcription level of the ergosterol synthesis pathway in the presence of artemisinin, and the transcription level of genes (*Erg1*, *Erg3*, *Erg9* and *Erg11*) was upregulated compared with that of the negative control DMSO (Figure 4C). This transcription level could be further enhanced by a high artemisinin concentration compared with the negative control DMSO. According to the former results that PDR1 mutant strains were resistant to artemisinin, we wanted to know whether artemisinin’s action on ergosterol synthesis was through PDR1 or not. We then measured the transcription level of *Erg* genes in the wild-type strain and PDR1 mutant strains incubated with artemisinin, and the results showed that the ERG genes were upregulated in the wild-type strain but not in PDR1 mutant strains (Figure 4D). These results suggested that PDR1 regulated *Cdr2* expression and thus influenced ergosterol synthesis under artemisinin incubation in fungi.

Further, we wanted to know whether artemisinin had an effect on the upstream pathway of the ergosterol synthesis or not. The ergosterol synthesis pathway could convert acetyl-CoA to ergosterol, but acetyl-CoA could be synthesized by pyruvate dehydrogenase (PDH) complex or acetyl coenzyme A synthetase (ACS) (Figure 4E). Thus, we measured the gene transcription level of acetyl-CoA synthesis with artemisinin incubation; PDH complex genes including *Pdh E1α*, *Pdh E1β*, *Pdh E2* and *Pdh E3* had no significant change compared with negative control (Figure 4F). *Acs1*, but not *Acs2*, was upregulated in the presence of artemisinin, and 20 μM artemisinin had a more obvious effect on *Acs1* expression than 10 μM artemisinin. Meanwhile, we measured the transcription level of *Acs1* in the wild-type strain and PDR1 mutations, and the results showed that *Acs1* expression had no significant change with artemisinin incubation in PDR1 mutant strains (Figure 4G). These data indicated that the ergosterol and acetyl-CoA synthesis pathways played an important role in artemisinin’s action in wild-type *C. glabrata* but not in artemisinin-resistant fungi, PDR1 mutant *C. glabrata*, in which PDR1 was a key regulator for artemisinin’s action on the ergosterol and acetyl-CoA synthesis.

### 3.5. Artemisinin Impairs Mitochondrial Function of Candida glabrata

**Figure 5:**
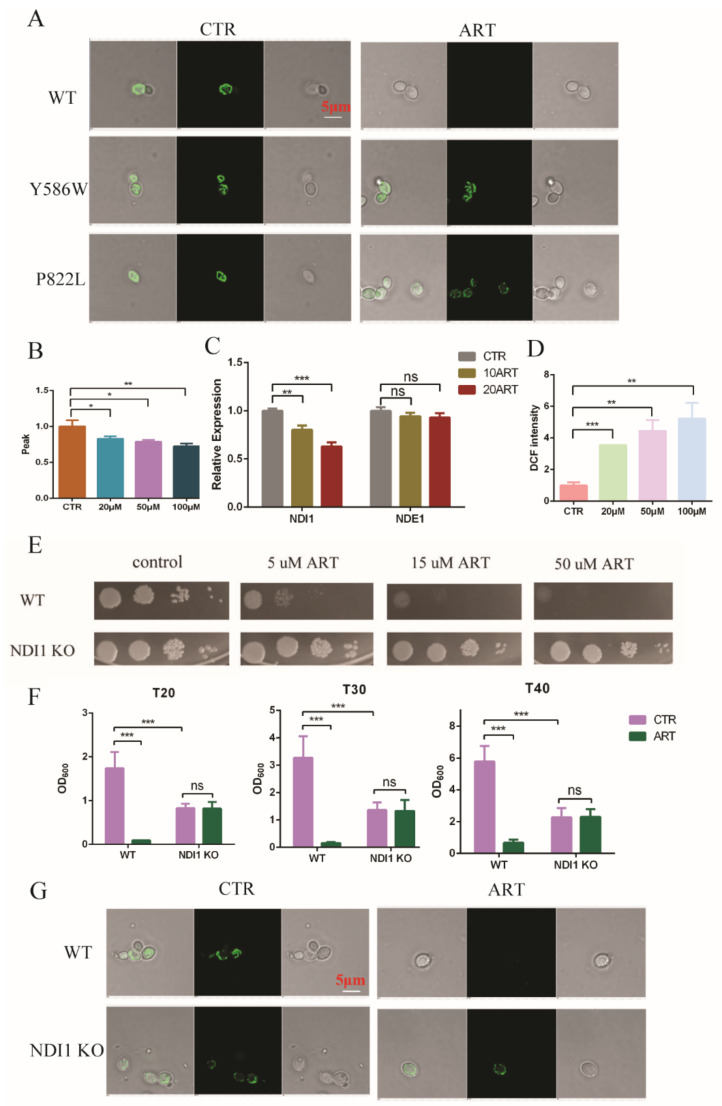
Mitochondria was disrupted with artemisinin. (**A**) Artemisinin depolarized *C. glabrata* mitochondrial membrane potential. (**B**) Artemisinin increased mitochondrial membrane viscosity of *C. glabrata*. (**C**) *Ndi1* expression was downregulated by artemisinin. (**D**) Artemisinin enhanced ROS level in *C. glabrata*. (**E**) *Ndi1* KO strain was resistant to artemisinin by spot assay. Four dots mean 10-fold serial dilutions of yeast cells. (**F**) The growth of *Ndi1* KO strain had no significant change with artemisinin in liquid medium. Data are mean ± SD of three independent experiments. Data were analyzed by GraphPad Prism. * *p*-value < 0.05, ** *p*-value < 0.005, *** *p*-value < 0.0005 and ns means no significant change (*p*-value > 0.05). (**G**) *Ndi1* KO strain had a lower mitochondrial membrane potential but could not be depolarized by artemisinin.

Based on the above results, we found that artemisinin inhibited the growth of *C. glabrata* by influencing the ergosterol and acetyl-CoA synthesis pathways via PDR1. Ergosterol is an important component of mitochondrial membrane, and thus gene deletion or overexpression in the ergosterol synthesis pathway has exhibited mitochondria dysfunction [31,32]. In addition, mitochondrial membrane potential was shown to depolarize with artemisinin in *S. cerevisiae* [33]. To investigate whether artemisinin could act on mitochondria in *C. glabrata* or not, we measured mitochondrial function. First, we examined the mitochondrial membrane potential using rhodamine probe-Rh123. We found that artemisinin depolarized the mitochondrial membrane potential of *C. glabrata*, but artemisinin-resistant strains—PDR1 mutants—showed no significant change under the same condition (Figure 5A). Then, we isolated fungal mitochondria and tested the mitochondrial membrane viscosity by measuring fluorescence spectra of merocyanine (MC540). The results are shown in Figure 5B, where the mitochondrial membrane viscosity was increased with the increase of artemisinin’s concentration. Next, we measured the transcription level of mitochondrial electron transport chain complex I as it was correlated to artemisinin’s sensitivity in *S. cerevisiae* [34]. *C. glabrata* had two type II NADH dehydrogenase-NDI1 and NDE1, and the results showed that *Ndi1* was downregulated in the presence of artemisinin, but *Nde1* showed no significant change compared with the negative control (Figure 5C). The mitochondria membrane is an important resource of reactive oxide species (ROS), and NADH dehydrogenase has been reported to reduce the production of ROS from mitochondria [35]. Hence, we finally examined the ROS level in the presence of artemisinin in *C. glabrata* and found that DCF’s (ROS probe) intensity was increased with the increase of artemisinin’s concentration (Figure 5D). These results suggested that mitochondrial function was disrupted by artemisinin, in which *Ndi1* might play an important role in artemisinin’s action.

To investigate *Ndi1* function in artemisinin’s effect, we knocked out *Ndi1* in *C. glabrata* and then measured the growth of fungi and the mitochondrial membrane potential in the presence of artemisinin. The growth of the wild-type strain was inhibited by the 5 μM artemisinin using spot assay, but *Ndi1* knocked-out (KO) strain was resistant to artemisinin even at 50 μM (Figure 5E). Then, we examined the OD_600_ of *C. glabrata* after 20 h, 30 h and 40 h of incubation with artemisinin using growth inhibition by a broth assay. The growth of the wild-type strain was inhibited by artemisinin obviously, but the *Ndi1* KO strain grew well with artemisinin (Figure 5F). Similar trends occurred with the mitochondrial membrane potential, and the results are shown in Figure 5G. Artemisinin depolarized the fungal mitochondrial membrane potential in the wild-type strain, but not in the *Ndi1* KO strain. The *Ndi1* KO strain had a lower mitochondrial membrane potential than that of the wild-type strain due to the disrupted electron transport chain. Taken together, these results suggested that *Ndi1* might be an important determinant of artemisinin’s susceptibility in fungi.

## 4. Discussion

In this study, the development of new antifungal drugs and a new drug action mechanism were reported. Artemisinin as a potential antifungal drug was selected from the FDA-approved medications library by high-throughput drug screening, and its inhibition on *C. glabrata* was confirmed by growth measurement. Then, we identified that PDR1 played an important role in artemisinin’s action using artemisinin resistant strains and molecular dynamics simulations. A further study showed that artemisinin affected the acetyl-CoA and ergosterol synthesis pathways through PDR1 regulation, leading to mitochondrial dysfunction. Finally, we found that artemisinin depolarized the mitochondrial membrane potential, increased the mitochondrial membrane viscosity and enhanced ROS levels in fungi. Our finding provided a new anti-*C. glabrata* drug and unveiled artemisinin’s action mechanism, which opened up a valuable avenue for the development of anti-*C. glabrata* agents from a known-drug-repurposing point of view. These findings offered a theoretical foundation for potential clinical treatment and shed new light and a better understanding of the mechanism of artemisinin’s action on other diseases. 

Artemisinin influenced transcription factor PDR1 leading to the growth inhibition of fungi. Artemisinin is sesquiterpene lactone derived from the traditional Chinese herb *Artemisia annua* L. [36]. Youyou Tu found that *A. annua* extracts had antimalaria activity in a mouse model infection in 1971 and was awarded the Nobel Prize in Physiology or Medicine for the discovery in 2015 [36,37]. Owing to artemisinin’s high efficacy and low toxicity, artemisinin combination therapy (ACT) is the first-line therapy for malaria treatment [38]. As for fungal infection treatment, Marcin et al. reported *A. annua* extracts had antifungal activity against *C. albicans*, *C. glabrata* and *S. cerevisiae* [39], and further, different candida species were used to study the cytotoxic action of artemisinin, among which *C. glabrata*, *C. guilliermondii*, and *C. parapsilosis* were more sensitive to artemisinin than other candida species [40]. However, artemisinin’s action mechanism was still unclear in fungi. In this work, we screened artemisinin as a potential antifungal drug from the FDA-approved drug library and found that PDR1 might be one of determinants of artemisinin’s action on fungi. Whether the *C. glabrata* growth inhibition by artemisinin was due to a fungistatic or fungicidal activity will be tested by a vitality cell assay in a further study.

Artemisinin influenced ergosterol’s biosynthesis leading to mitochondrial dysfunction through PDR1 regulation. *A. annua* extracts and artemisinin were reported to be transported by *Pdr5* in *S. cerevisiae*, which was regulated by transcription activator-PDR1 [39]. Moreover, artemether enhanced fluconazole efficacy via *Pdr5*, and thus stimulated intracellular fluconazole accumulation [41]. In this work, the transcription level of *CgCdr2*—homologue protein of *ScPdr5*—was upregulated by artemisinin incubation, possibly causing intracellular artemisinin accumulation in fungi. Further, *CaCdr1* (homologue protein of *ScPdr5*) was related to ergosterol content in *C. albicans* [29]. Ergosterol synthesis pathway genes such as *Erg1* and *Erg9* could be upregulated by artemisinin incubation in *C. albicans* [42], and these findings were also confirmed in our study. Meanwhile, the transcription level of ergosterol synthesis genes had no significant change with artemisinin incubation in PDR1 mutants. Ergosterol is an important component of the mitochondrial membrane, and mitochondria play an important role in artemisinin’s action on malaria, yeast and cancer cells. First, artemisinin as a antimalarial drug depolarized the extracted mitochondrial membrane potential of malaria but not mammalian cells [33]. Another study reported that artemisinin disrupted the mitochondrial membrane potential in *S. cerevisiae*, resulting in mitochondrial dysfunction [34]. Furthermore, the synthesized mitochondrial-targeting artemisinin derivatives through conjugating triphenylphosphonium to artelinic acid sharply increased its anticancer activity [43]. However, which genes or pathways influenced mitochondria function leading to cell death has not be well-studied. Azoles are the main classes of clinical antifungal agents, and cgPDR1 has been reported to be required for the azole resistance by mitochondrial deficiency [44], but how cgPDR1 regulates the cellular pathway and impacts mitochondrial function was previously unknown. In this study, we showed that PDR1 regulated the lipid synthesis and then impacted the mitochondrial membrane viscosity and depolarized the mitochondrial membrane potential. Further, we analyzed which mitochondria-related genes participated in artemisinin’s action. NADH dehydrogenase of the mitochondrial electron transport chain was correlated with artemisinin’s action in *S. cerevisiae* [34] and *Aspergillus fumigates* [45]. In our study, *Ndi1* knockout strain was resistant to artemisinin and thus restored the mitochondrial membrane potential depolarization.

In conclusion, artemisinin as a potential antifungal drug had an effect on the lipid synthesis pathway, leading to mitochondrial dysfunction by transcription receptor-PDR1. This discovery still needs further studies in the future. For example, the antifungal activity of artemisinin needs to be confirmed in the clinical isolates or fluconazole-resistant *C. glabrata* strains. The mitochondrial function of *C. glabrata* with artemisinin incubation such as mitochondrial fusion and fission may be investigated in the future. Furthermore, due to the antifungal drug resistance and side-effect of clinical antifungal agents, developing a combination therapy of current antimycotics with artemisinin or other natural products is required in the future. Artemisinin may be a potential clinical drug to treat individuals coinfected with malaria and fungi because of its multiactivity. On the other hand, artemisinin and its derivatives showed efficacy against a lot of diseases, such as human cancers [46], leishmaniasis [47], tuberculosis [48] and SARS-CoV-2 [49]. Based on this work, a further study about artemisinin’s action mechanism on other diseases will be required.

## 5. Conclusions

Fungal pathogens especially candida species pose a serious threat to human health. To deal with this, a few therapies for candida treatment are available, but the side-effects of clinical antifungal agents and pathogens resistance create an urgent need to develop new antifungal drugs. The antimalarial drug artemisinin selected from the FDA-approved drug library has potential as a therapeutic agent for *C. glabrata*. Transcription factor-cgPDR1 plays an important role in artemisinin action through regulating the drug efflux pump and the ergosterol biosynthesis pathway, resulting in mitochondrial dysfunction. This discovery described here sheds new light on understanding artemisinin’s multiaction mechanism and developing new antifungal agents.

## Figures and Tables

**Table 1:** Antifungal drugs in high-throughput screening.

Class	Drug	Inhibition at 12 h (%)	Inhibition at 24 h (%)
Polyenes	Amphotericin B	68.05	48.47
Echinocandins	Caspofungin Acetate	81.68	84.28
Imidazoles	Bifonazole	62.94	82.78
Triazoles	Fluconazole	63.79	78.03
Antiseptics	Benzethonium Chloride	85.07	86.41

**Table 2:** Effect of point mutations on the binding energy of artemisinin interaction with PDR1.

Amino Acid	Binding Free Energy (kcal/mol)	Amino Acid	Binding Free Energy (kcal/mol)	Binding Free Energy Change (%)
R376	−21.34	R376W	−10.04	52.95
Y584	−29.71	Y584C	−20.09	32.38
P822	−40.59	P822L	−30.55	24.74
D1082	−25.11	D1082G	−18.41	26.68

## Data Availability

Data are contained within the article.

## Supplementary Materials

The following supporting information can be downloaded at: https://www.mdpi.com/article/10.3390/antiox11101855/s1, Table S1. *C. glabrata* strains in this study, Table S2. qPCR primers used in this study.
