# Supplementary material for: Artemisinin Targets Transcription Factor PDR1 and Impairs Candida glabrata Mitochondrial Function
Source: Antioxidants (Basel). 2022 Sep 20;11(10):1855. doi: 10.3390/antiox11101855 (PMC9598568; doi:10.3390/antiox11101855)
Supplement: Supplementary file 1 [file antioxidants-11-01855-s001.zip › antioxidants-1882129-supplementary.pdf]

Supplementary Materials

Artemisinin targets transcription factor PDR1 and impairs *Candida glabrata* mitochondrial function

Pan Zhu[1,2] , Chaoping Yue[3], Xin Zeng[3] and Xiulai Chen[1]

[1]State Key Laboratory of Food Science and Technology, Jiangnan University, Wuxi 214122, China

[2]State Key Laboratory of Membrane Biology, School of Life Sciences, Tsinghua University, Beijing 100084, China

[3]Anhui Province Key Laboratory of Pollutant Sensitive Materials and Environmental Remediation, Huaibei Normal University, Huaibei 235000, China

Correspondence:

Xiulai Chen, State Key Laboratory of Food Science and Technology, Jiangnan University, Wuxi 214122, China. Email: xlchen@jiangnan.edu.cn

Table S1. *C. glabrata* strains in this study.

Stains	Genotype	Reference
C. glabrata ATCC 2001	Wild type	[1]
C. glabrata ATCC 55	*his3Δ trp1Δ ura3Δ*	[2]
Ndi1 KO	*his3Δ trp1Δ ura3Δ ndi1::His3*	This study
$PDR1^{R376W}$	*his3Δ trp1Δ ura3Δ pdr1::His3 (pY14-PDR1* ^{R376W})	This study
$PDR1^{Y584C}$	*his3Δ trp1Δ ura3Δ pdr1::His3 (pY14-PDR1* ^{Y584C})	This study
$PDR1^{P822L}$	*his3Δ trp1Δ ura3Δ pdr1::His3 (pY14-PDR1* ^{P822L})	This study
$PDR1^{D1082G}$	*his3Δ trp1Δ ura3Δ pdr1::His3 (pY14-PDR1* ^{D1082G})	This study

Table S2. qPCR primers used in this study.

Primers	Sequence
ACTIN qPCR-F	ACCGCTGCTCAATCTTCC
ACTIN qPCR-R	TCCTTACGAACATCAACATCAC
ERG1 qPCR-F	ACGATGTTGTCCTATTGAT
ERG1 qPCR-R	CGAAGTGGTAATCTAGCA
ERG3 qPCR-F	CTTCGGTCTGCTATTGTA
ERG3 qPCR-R	ACATCTGGTTCTTCAAGTA
ERG9 qPCR-F	GACCAACATTATCAGAGA
ERG9 qPCR-R	CTTCGTAGCATATTCAGA
ERG11 qPCR-F	TATGGTCGCCTTG CCATT
ERG11 qPCR-R	GACCCATGGGATCCAGTAGA
ACS1 qPCR-F	GTGTCGACAGACACGCTTTG
ACS1 qPCR-R	GAGAAACCTGCGAAGACGAC
ACS2 qPCR-F	CGCTGTGGTTGGTATCAATG
ACS2 qPCR-R	AGCGAATGGACCAATTTCAC
PDH E1α qPCR-F	CTCCAACCAAGGTCAGGTGT
PDH E1α qPCR-R	AGCTTGGTAGACAGCCAGGA
PDH E1β qPCR-F	AACTTGCGTTCCATCAGACC
PDH E1β qPCR-R	GCACCGGTGACTCTTTGAAT
PDH E2 qPCR-F	GGATGCTGGTGGATCTCAGT
PDH E2 qPCR-R	CTTCTCGACATCGGCCTTAG
PDH E3 qPCR-F	CCGTTATCGGTGGTGGTATC
PDH E3 qPCR-R	TCGGCAGAGACGACCTTAGT
NDI1 qPCR-F	CCTATGCTCAATCTGTCTT
NDI1 qPCR-R	GAATGGTCTCCTCTGTAAC
NDE1 qPCR-F	CCTACATTGGCTCTGAAC
NDE1 qPCR-R	TGACATACACATTGCTAAGT

References

1. Gregori, C.; Schüller, C.; Roetzer, A.; Schwarzmüller, T.; Ammerer, G.; Kuchler, K., The high-osmolarity glycerol response pathway in the human fungal pathogen Candida glabrata strain ATCC 2001 lacks a signaling branch that operates in baker's yeast. *Eukaryotic cell* **2007,** 6, (9), 1635-45.

2. Roetzer, A.; Gregori, C.; Jennings, A. M.; Quintin, J.; Ferrandon, D.; Butler, G.; Kuchler, K.; Ammerer, G.; Schuller, C., Candida glabrata environmental stress response involves Saccharomyces cerevisiae Msn2/4 orthologous transcription factors. *Mol Microbiol* **2008,** 69, (3), 603-20.
